# NRITLD Protocol for the Management of Patients with COVID-19 Admitted to Hospitals

**Published:** 2020-11

**Authors:** Majid Marjani, Payam Tabarsi, Afshin Moniri, Seyed Mohammadreza Hashemian, Seyed Alireza Nadji, Zahra Abtahian, Majid Malekmohammad, Arda Kiani, Behrooz Farzanegan, Alireza Eslaminejad, Atefeh Fakharian, Jalal Heshmatnia, Atefeh Abedini, Sharareh Seifi, Fatemeh Yassari, Maryam Sadat Mirenayat, Mitra Rezaei, Hakimeh Sheikhzade, Zargham Hossein Ahmadi, Farzaneh Dastan, Mohsen Sadeghi, Somayeh Lookzadeh, Mihan Porabdollah, Elham Askari, Parvaneh Baghaei, Babak Mansourafshar, Alireza Jahangirifard, Maryam Vasheghani, Mojtaba Mokhber Dezfuli, Mohammad Varahram, Hamidreza Jamaati, Davood Mansouri, Alireza Zali, Ali Akbar Velayati

**Affiliations:** 1 Clinical Tuberculosis and Epidemiology Research Center, National Research Institute of Tuberculosis and Lung Diseases, Shahid Beheshti University of Medical Sciences, Tehran, Iran,; 2 Virology Research Center, National Research Institute of Tuberculosis and Lung Diseases, Shahid Beheshti University of Medical Sciences, Tehran, Iran,; 3 Tracheal Diseases Research Center, National Research Institute of Tuberculosis and Lung Diseases, Shahid Beheshti University of Medical Sciences, Tehran, Iran,; 4 Chronic Respiratory Disease Research Center, National Research Institute of Tuberculosis and Lung Disease (NRITLD), Shahid Beheshti University of Medical Sciences, Tehran, Iran,; 5 Lung Transplant Research Center, National Research Institute of Tuberculosis and Lung Diseases, Shahid Beheshti University of Medical Sciences, Tehran, Iran,; 6 Mycobacteriology Research Center, National Research Institute of Tuberculosis and Lung Diseases, Shahid Beheshti University of Medical Sciences, Tehran, Iran,; 7 Department of Clinical Immunology and Infectious Diseases, National Research Institute of Tuberculosis and Lung Diseases, Shahid Beheshti University of Medical Sciences, Tehran, Iran,; 8 Research Center for Neurosurgery and Functional Nerves, Shahid Beheshti University of Medical Sciences, Tehran, Iran.

## INTRODUCTION

In December 2019, the first case of coronavirus disease 2019 (COVID-19), was reported from China. An emerging infectious disease caused by a new strain of coronavirus, namely severe acute respiratory syndrome coronavirus-2 (SARS-CoV-2) led to a global health problem and pandemic (1).

By 28 May 2020, the total number of laboratory-confirmed cases of COVID-19 surpassed 5,701,337, with 357,688 reported deaths worldwide (2). Iran reported its first confirmed cases of COVID-19 on the 19 February 2020 in Qom, Qom Province (3), and by 29 May 2020, a total of 143,849 confirmed cases and 7627 deaths were reported (2).

More than 300 active clinical trials concerning agents and therapeutic measures potentiality effective in the treatment of COVID-19 are underway. However, no proven effective therapies for this virus are supported by any currently existing randomized clinical trials (RCTs) (4).

### Scope of this protocol and ethical issues

The guideline released by the National Research Institute of Tuberculosis and Lung Diseases (NRITLD), Masih Daneshvari Hospital COVID-19 Expert Group, may help the clinicians caring for patients admitted to hospitals with confirmed or suspected SARS-CoV-2 infection. Care of mild and outpatient cases and interventions for infection control are beyond the scope of the present paper.

Due to the emerging aspect of COVID-19 and its evolving knowledge, any recommendation may change in the future. Therefore, clinicians should update their information in short periods. On the other hand, adherence to national and local guidelines is mandatory, and prescription of any drug out of national protocols should be done just under clinical trials that obtained informed consent from patients and were approved by an ethics committee.

### Classification of severity

The severity of respiratory problems in COVID-19 is defined as follows:

**Mild:** Symptomatic patients without pulmonary infiltration and peripheral capillary oxygen saturation (SpO_2_) of >93% in ambient air.
**Moderate:** Patients with pulmonary infiltration and SpO_2_ of >93% in ambient air.
**Severe:** Patients with the respiratory rate (RR) of ≥30 breath/minute, SpO_2_ ≤93% in ambient air, or pulmonary infiltrates in more than 50% of the lung field, not in a critical state.
**Critical:** Patients admitted to the intensive care unit (ICU); patients who need high flow oxygen with a nasal cannula, noninvasive ventilation, or mechanical ventilation; the ones with acute respiratory distress syndrome (ARDS) or shock (5,6).

### Indications of a virologic assay

Virologic assay by reverse transcriptase polymerase chain reaction (RT-PCR) is recommended for any patient with an indication of hospital admission. It is also necessary for symptomatic health care workers taking care of suspected or confirmed cases of COVID-19.

The highest accuracy for the detection of SARS-CoV-2 can be obtained by using the lower respiratory tract specimens. However, the collection of sputum and bronchoalveolar lavage puts the healthcare workers at a higher risk due to aerosols formation. Nasopharyngeal or oropharyngeal swab/wash is the routine method to obtain a specimen for testing. It is reported that the detection of the COVID-19 virus with oropharyngeal swabs was less frequent than nasopharyngeal ones (7).

In Iran, due to restrictions on obtaining nasopharyngeal swabs, the oropharyngeal swabs or washing comprise the majority of respiratory tract specimens. Clinicians should consider that a single negative RT-PCR is not sufficient to exclude COVID-19, especially if the clinical suspicion is high. Therefore, they should consider test repetition and eventually rely on compatible symptoms, exposure history, and typical CT imaging features for the diagnosis of COVID-19 (8).

At present, based on the current evidence, the world health organization (WHO) recommends the utilization of serologic tests only in research settings and not for clinical decision-making (9).

### Indications of imaging study

1. Normal host symptomatic cases with SpO_2_ of ≤93% in ambient air
2. Immunocompromised patients with any symptom (mainly, but not limited to, fever and hypoxemia) suspected of COVID-19
3. Any febrile patients with the risk factors of severe disease (BMI ≥40 kg/m^2^ , hypertension, chronic pulmonary/cardiovascular disease, and diabetes mellitus) suspected of COVID-19

Although the most sensitive modality is the lung CT-scan (10), plain chest x-ray (CXR) may be adequate in normal hosts. For immunocompromised patients, a CT-scan of the lung is highly recommended. Routine use of CT-scan, as a screening measure, among asymptomatic cases should highly be deferred (11).

### Indications of admission

Indications of in-hospital management are: RR ≥30 breath/minute; SpO_2_ ≤93% in ambient air, any abnormalities in CXR, considerable infiltration in the lung CT-scan, decreased level of consciousness, shock, and clinicians’ judgment (e g, in the case of alone patients or oral intolerance).

Admission to hospital is not necessary for patients with small and tiny pulmonary infiltration in the lung CT-scan unless other indications exist.

Mild cases should be managed as an outpatient, but patient education is necessary to come back in the case of deterioration.

### Protocol of treatment

### Moderate cases

#### - Chloroquine

Chloroquine (in our setting, hydroxychloroquine sulfate), as monotherapy, is recommended for outpatient therapy. It can be prescribed for symptomatic high-risk groups without pneumonia; two tablets b.i.d for the first day and then one tablet b.i.d for 5–10 days (12). In the hospital, when lopinavir/ritonavir is the basic regimen, (13) it is recommended to prescribe hydroxychloroquine just on the first day with a dosage of two tablets b.i.d in order to prevent serious interactions.

In a recent observational study, the administration of hydroxychloroquine was not associated with the decrease of intubation or mortality rates (14). Therefore, recommendations for the prescription of this drug to treat COVID-19 may change in the future.

#### - Lopinavir/ritonavir

Lopinavir/ritonavir, sold as Kaletra®, is the basic regimen for the cases admitted to hospitals, recommended by the Iranian National COVID-19 Scientific Program. Its efficacy is controversial (4), and in a recent study, no benefit was observed with lopinavir-ritonavir treatment beyond the standard care in hospitalized adult patients with severe COVID-19 (15). It is prescribed as two 200/50 mg tablets b.i.d. Kaletra® causes critical adverse effects and interacts with other drugs. The most serious one is the QT interval prolongation and life-threatening arrhythmia (4). Concomitant administration of drugs with the QT prolongation effects, such as fluoroquinolones, methadon, ondansetron, and metoclopramide, should be avoided in order to prevent arrhythmia. Concomitant administration of Kaletra® with rivaroxaban, lovastatin, simvastatin, colchicine, cisapride, and sildenafil to treat pulmonary artery hypertension is contraindicated (16). It is necessary to get the baseline ECG, and if the corrected QT interval is longer than 500 msec, the drug should be discontinued. For the QT intervals between 450 and 500 msec, the daily repetition of ECG is recommended. Correction of serum electrolytes, such as potassium >4 mEq/L and magnesium >3 mEq/L, is recommended to decrease the chance of arrhythmia (16).

The most frequent problem with Kaletra® is gastrointestinal upset. Taking tablets with food may decrease the chance of this problem (17).

Kaletra® should be prescribed for minimally seven days, but this period can be extended to 14 days (4).

#### - Remdesivir (as a clinical trial) (IRCT20171122037571N2)

The preliminary findings support the administration of remdesivir to treat hospitalized patients with COVID-19, especially before the disease progression leading patients to require mechanical ventilation (18). Remdesivir is administered intravenously as a 200-mg loading dose on day 1, followed by a 100-mg maintenance dose administered daily for of 5 to 10 days (19,20).

The efficacy of remdesivir is under investigation by the WHO in the Solidarity Trial. The Solidarity Trial (an international trial conducted by WHO) compares four treatment options (remdesivir, lopinavir/ritonavir, lopinavir/ritonavir with interferon beta-1a, and hydroxychloroquine) against the standard of care to assess their relative effectiveness against COVID-19 (21).

#### - Favipiravir (as a clinical trial) (IRCT20151227025726N14)

Favipiravir, sold as Avigan®, is an antiviral agent administered to treat influenza before COVID-19 pandemic (22) and is a promising agent to treat COVID-19 (23). Multiple clinical trials are ongoing in the world to evaluate its efficacy among patients with COVID-19 (4).

Favipiravir can be used as the first-line antiviral agent (alternative to Kaletra®) or the secondary agent when Kaletra® fails, and the patient condition worsens (both of them as clinical trials).

Different dosages of favipiravir are used in various trials. The dose of 1600 mg b.i.d on the first day, followed by 600 mg b.i.d for seven days is recommended (23).

Favipiravir is a teratogen; therefore, its administration is contraindicated in pregnancy. Limited data are available about its administration in renal insufficiency, but it seems that the dose adjustment is not necessary. Increased exposures are observed in Child-Pugh class A to C, and dose adjustment should be considered in Child-Pugh C (4).

#### - Umifenovir (as a clinical trial) (IRCT20151227025726N15)

Umifenovir, sold as Arbidol®, can be used as an alternative to Kaletra® (24), but its prescription should be limited to approved clinical trials. Arbidol® is available as 100 mg capsules, and the dosage is 200 mg TDS for seven days. No dose adjustment is necessary in renal failure, and there is no specific recommendation in the case of hepatic impairment (4).

#### - Oseltamivir and Ribavirin

As the recommendation of the National Scientific Committee of COVID-19, and termination of seasonal influenza circulation in the community, oseltamivir is no longer prescribed. Also, ribavirin is withdrawn from the COVID-19 drug regimen in Iran.

### Severe cases

#### - Interferon beta 1-a (as a clinical trial) (IRCT 20151227025726N12)

Interferon beta 1-a can be added to the basic regimen in the context of an approved clinical trial (4,21,25–27). Inclusion criteria are:

- Bilateral pulmonary involvement
- At rest SpO_2_ of <90% in ambient air

Exclusion criterion is:

- Intubation

The recommended dose is 12 million units subcutaneous, every other day for three to five doses (28).

#### - Steroid

A recent preliminary report of the RECOVERY Trial shows that a low dose of dexamethasone reduces the risk of death in patients with COVID-19 requiring supplemental oxygen (29). We recommend 4 mg dexamethasone b.i.d for 10 days if SpO_2_ remains less than 90%.

#### - IVIG (as a clinical trial)

Intravenous immunoglobulin (IVIG) preparation can be added to the basic regimen based on an approved clinical trial (4,30).

Inclusion criteria:

- Bilateral pulmonary infiltration
- At rest SpO_2_ of <90% with fraction of inspired oxygen (FiO_2_) >30%–40%

IVIG dosage is 25 g/day for three days.

#### - Tocilizumab (as a clinical trial) (IRCT 20151227025726N13)

For patients under 65 years old with positive PCR results and SpO_2_ <90%, the quantification of plasma interleukin 6 (IL-6) level is recommended. If the IL-6 level is higher than 7 pg/mL, the prescription of tocilizumab, sold as Actemra®, should be considered in the context of an approved clinical trial. For this purpose, an expert team should discuss the case and decide.

Inclusion criteria for tocilizumab administration are:

- Age between 18 and 65 years
- Confirmation of COVID-19 by PCR
- Critical or severe cases with minimally one of the following conditions:
Admission to ICU
At rest RR of ≥30 breath/minute
SpO_2_ of <90% in ambient air
PaO_2_/FiO_2_ of <300
Serum IL-6 level of ≥7 pg/mL

Exclusion criteria are:

- Acute or chronic renal failure
- Rise of liver enzymes more than 5x upper limit of normal (ULN); or more than 3x ULN and symptomatic; or severe liver insufficiency with class C Child-Pugh score.
- Pregnancy or nursing mother
- Any active infection other than COVID-19
- Active peptic ulcer disease
- Intubation

Before the administration of the drug, latent TB and viral hepatitis should be evaluated. Also, informed patient consent is necessary. Tocilizumab is prescribed as a single dose of 400 mg drug diluted in 100 mL normal saline via IV infusion within one hour. Close monitoring is essential during the infusion (4,31– 33).

#### - Convalescent plasma therapy (as a collaborative center in a multi-institutional trial) (IRCT20200325046860N1)

Convalescent plasma prepared by plasmapheresis from recovered cases of COVID-19 is used to treat severe cases unresponsive to other measures (34). Although promising, its safety is a concern and should be administered under strict monitoring (35). RCTs to evaluate convalescent plasma for the treatment of COVID-19 are underway. The process of donation, collection, preparation, and testing is very complex and should be performed just by an authorized organization (in Iran, the Iranian Blood Transfusion Organization).

Candidates for plasma therapy are confirmed or highly suspected cases of COVID-19 who are hypoxemic (SpO_2_ <90% in ambient air) with no improvement after minimally 48 hours of conventional treatment. Transfusion of one unit of plasma (500 to 650 mL) is performed within four hours under careful monitoring for transfusion reactions. In the case of the clinician request, one more unit can be transfused after 24 hours for unresponsive cases (34,36, 37).

### ICU Care

Criteria for the ICU admission of patients with COVID-19 are:

1. SpO_2_ of ≤85% or partial pressure of arterial oxygen (PaO_2_) of ≤60 mmHg unresponsive to appropriate oxygen therapy
2. Severe respiratory distress
3. Hemodynamic instability
4. Acid-base disturbance
5. Other ICU admission criteria (38).
  - Respiratory support strategy

There are two types of hypoxic patients with COVID-19 pneumonia and different mechanisms and pathophysiology, clearly distinguishable by the lung CT-scan. In the first group, lung compliance is nearly normal, without ARDS, and in the second, the compliance is low with ARDS. In type 2 patients, in addition to viral pneumonia, they likely have self-induced lung injury due to vigorous inspiratory efforts leading to highly negative intrathoracic pressures and increased edema. Therefore, early intubation and subsequent invasive mechanical ventilation in rapidly progressing respiratory distress can diminish the change from type 1 to type 2 and decrease the risk of self-induced lung injury (39,40).

#### - Noninvasive ventilation and high flow nasal therapy

We recommend NIV versus early intubation in selected patients with COVID-19 that can tolerate masks, are cooperative, and have no signs of severe respiratory distress. Whenever any intolerance or disadvantages of NIV are suspected, the strategy is changed to the early invasive ventilation protocol.

The main concern about NIV is the aerosol formation and hazard for health care staff (41). Ideally, NIV should be performed in the ICU setting with a negative pressure system, the air passing through the high-efficiency particulate air filters, and exhaust directly to the exterior.

NIV needs an ICU team capable of working with noninvasive machines and familiar with mask technologies, such as helmet and full-face masks and new modes of noninvasive ventilation.

#### - Invasive mechanical ventilation

If severe respiratory distress is present, or when the hypoxemia is nonresponsive to NIV, endotracheal intubation should be considered to prevent self-induced lung injury. We recommend the application of the low tidal volume strategy to reduce ventilator-induced lung injury (40); PEEP levels 8–10 cm H_2_O in type 1 patients and 14–15 cm H_2_O in type 2 cases, and plateau airway pressure maximally 30 cm H_2_O. Then the RR is increased to maximally 30 breath/minute if necessary, which is the mainstay of the lung-protective ventilation.

Also, ventilation in the prone position is recommended as a rescue maneuver in type 1 and as a long-term treatment in type 2 patients (39). An early tracheostomy with preferred the mini-surgical percutaneous dilatational tracheostomy method is recommended (42).

#### - Steroid

For the patients admitted to ICU with any stage of ARDS, the prescription of corticosteroids is recommended to decrease the inflammation. The recommended dose is 20 mg dexamethasone daily from day 1 to 5, then 10 mg daily from day 6 to 10. This approach is applied based on the study by Villar et al., (43) in the rapid progression of ARDS in patients with COVID-19 in order not to lose the time.

#### - Extracorporeal membrane oxygenation

In patients under mechanical ventilation, with PaO_2_/FiO_2_ <80 for six hours or PaO_2_/FiO_2_ <50 for three hours, despite having high positive end-expiratory pressure and neuromuscular blockage, it is planned for ECMO. Transthoracic echocardiography is performed; if left ventricular ejection fraction is >50% the veno-venous ECMO, and if <50% or in case of hemodynamic instability, the veno-arterial ECMO are planned.

The drainage cannula is inserted by the close Seldinger maneuver in the left or right femoral vein, and the return cannula by the same technique in the right internal jugular vein. The position of the cannula is checked by CXR, and the efficacy of the system is evaluated by increasing arterial oxygen saturation and PaO_2_. All patients receive a continuous dose of intravenous heparin with the activated partial thromboplastin time (aPTT) of 50–70 seconds. The aPTT is checked every six hours daily.

### Other strategies

Rehydration and appropriate nutritional support is recommended.

Also, a standard dose of venous thromboembolism prophylaxis with low molecular weight or unfractionated heparin is recommended for all the patients with COVID-19 admitted to hospitals. For ARDS cases and patients admitted to ICU, an escalated dose of heparin (half of a therapeutic dosage) is preferred (44).

Indication of stress ulcer prophylaxis is similar to those of other patients admitted to hospitals.

### Hospital discharge planning

The decision to discharge patients is clinical-based. Overall admission duration of minimally seven days is recommended.

The following criteria are recommended for discharge planning:

- Being afebrile for 72 hours without receiving antipyretic drugs
- A significant improvement in imaging studies (CT-scan or CXR as the physician preference)
- SpO_2_ of ≥93% in ambient air
- Stability of vital signs (blood pressure, RR, and pulse rate)
- Tolerance of oral intake

PCR testing before discharge is necessary, but the negative result PCR is not mandatory for patient discharge, although it is preferable.

The patient has to be isolated in his or her house for minimally two weeks. Doctors and nurses should train any patient before discharge from the hospital both verbally and by providing pamphlets, especially in the infection control measures.

### Follow-up

If follow-up imaging is necessary, it recommends 6–8 weeks after discharge. The selection of the modality is on the basis of extension of involvement in the previous imaging studies.

### Prophylaxis

Currently, there is no evidence of any clinical trial supporting any prophylactic regimen (4). We do not recommend the use of any drug for the prevention of COVID-19 by health care workers or others.

**Table 1.** Drug dosage and schedule

**Drugs**	**Dosage**	**Duration**	**Comments**
Hydroxychloroquine sulfate	400 mg/PO/bid 1^st^ day then 200 mg/PO/bid	7–14 days	In concomitant usage with Kaletra, just loading dose (1^st^ day) is recommended.
Lopinavir/ritonavir (Kaletra)	200/50 mg II/PO/bid	7–14 days	The basic regimen as Iranian protocol.
Favipiravir (Avigan)^*^	1600 mg/po/bid 1^st^ day then 600 mg/bid	7 days	
Umifenovir (Arbidol)^*^	200 mg t.i.d	7 days	
Remdesivir^*^	200 mg 1^st^ day then 100 mg daily	5–10 days	
Interferon β 1 a	12 MU/SC/every other day	5 doses	In severe cases
Dexamethason	4 mg / IV / b.i.d	10 days	In severe cases
IVIG^*^	25 gr / IV infusion / daily	3 days	In severe cases
Tocilizumab (Actemra)^*^	8 mg/kg (400 mg) / slow IV infusion	Single dose	Can be repeated after 12 – 24 hours

* Just to be used as approved clinical trials
